# Antiretroviral Use in Resource-Poor Settings: Modeling Its Impact

**DOI:** 10.1371/journal.pmed.0030179

**Published:** 2006-03-14

For many people in the developed world, a diagnosis of HIV/AIDS is no longer a death sentence. Since the introduction in the 1990s of effective antiretroviral therapy (ART)—combinations of three or four drugs that interfere with different stages of the HIV life cycle—HIV/AIDS mortality rates have dropped by 50%–70% in affluent countries. By contrast, in developing countries, at least 6 million people need immediate access to ART but fewer than 10% of them get it. In 2005, 3 million people in poor countries died from HIV/AIDS. To improve this situation, the World Health Organization (WHO), the Joint United Nations Programme on HIV/AIDS (UNAIDS), and other international bodies are working toward providing universal access to ART for all those who need it by 2010. In addition to reducing AIDS morbidity and mortality, the hope is that this strategy will also reduce HIV/AIDS prevalence, because sexual transmission of HIV is more likely if the partner who is HIV-positive has a high viral load.

Many challenges need to be overcome to achieve universal access to ART, not least of which is determining how to maximize the benefits of ART to patients and their communities in resource-poor settings. Regional differences in health-care facilities, local changes in sexual behavior in response to treatment, and many other factors can alter how ART affects both HIV transmission rates and HIV/AIDS mortality. Ideally, the best strategy for each setting would be determined through large-scale randomized trials of different approaches—for example, the time at which treatment is initiated relative to the time of infection—with HIV prevalence and HIV/AIDS–related mortality as primary endpoints. However, such trials are lengthy and costly, so researchers and policy makers are also using mathematical models to explore the impacts of different treatment and monitoring strategies. Rebecca Baggaley and colleagues now describe a new approach to modeling the impact of ART in resource-poor settings. Their model predicts that HIV epidemics in sub-Saharan Africa will not be controlled through ART alone, even if universal access is achieved. Additional prevention methods such as counseling patients and their communities about safe sex are essential. Without them, their results suggest, access to ART is likely to increase HIV/AIDS prevalence.

The researchers' deterministic model of HIV transmission incorporates ART and stratifies infection progression into four different stages (primary infection, incubation, pre-AIDS, and AIDS), each of which is associated with a different degree of infectiousness. In effect, the model is a complex flowchart through which patients move inexorably as they become infected and receive treatment—which can fail (virologic failure) or succeed (long-term viral suppression)—or from which they can withdraw. Sexual behavior and changes in sexual behavior in response to HIV/AIDS and ART is also plugged into the model—people treated with antiretrovirals often become more sexually active as they begin to feel better. Effective counseling, on the other hand, can increase safe-sex practices. To turn this flowchart into predictions of how HIV epidemics in sub-Saharan Africa might develop over time given different ART strategies and, for example, the availability of diagnostic laboratories to monitor the immune status and viral load of individuals with HIV, the researchers used published estimates of relevant parameters such as the fraction of patients that drop out at each stage of treatment and the transmission probability per sexual partnership for patients in whom ART failed.

Baggaley and her colleagues make several predictions. They suggest, for example, that unlimited ART provision initiated once patients have developed AIDS will increase the prevalence of infection (because the patients live longer and become sexually active again), a worrying result given that one aim of the universal access initiative is to reduce HIV infection rates. Furthermore, although different coverage levels in this scenario will not affect the years of life gained per person-year of treatment, increased coverage will increase the emergence and spread of drug resistance. If pre-AIDS patients are treated as well, the researchers predict that additional infections will be averted per person-year of treatment, but the effect will be small and highly dependent on how pre-AIDS patients change their sexual behavior in response to ART.

As with all modeling exercises, this new model includes many assumptions that may limit its applicability in the “real world.” For example, it includes only first-line triple-therapy ART and does not allow for second-line therapy if one drug regimen fails; it does not consider the sexual behavior of people who don't know they are infected with HIV; and it does not allow for a reduction in the quality of ART programs as coverage increases, a likely problem in countries with limited resources. Nevertheless, the model's predictions sound a warning: ART is not likely to function as a direct method for transmission prevention even when coverage is high. Counseling of patients and their communities to promote safe sexual practices must accompany ART provision. Difficult decisions regarding the allocation of finite resources will have to be made as ART is rolled out in resource-poor countries, conclude the researchers, decisions that can best be made by combining mathematical modeling with data from early programs as they become available.

